# Effect of metformin use on clinical outcomes and serum urate in gout patients with diabetes mellitus: a retrospective cohort study

**DOI:** 10.1186/s41927-022-00261-3

**Published:** 2022-05-31

**Authors:** Frouwke Veenstra, Lise M. Verhoef, Merel Opdam, Alfons A. den Broeder, Wing-Yee Kwok, Inger L. Meek, Cornelia H. M. van den Ende, Marcel Flendrie, Noortje van Herwaarden

**Affiliations:** 1 Department of Rheumatology, Sint Maartenskliniek, Hengstdal 3, Ubbergen, 6574 NA Nijmegen, The Netherlands; 2 Rheumatology, Radboud University Medical Center, Radboud Institute for Health Sciences, Nijmegen, The Netherlands; 3 Department of Rheumatology, Rijnstate Hospital, Arnhem, The Netherlands; 4 Department of Rheumatology, Radboud University Medical Center, Nijmegen, The Netherlands

**Keywords:** Gout, Diabetes Mellites, Metformin, mTOR inhibition

## Abstract

**Objective:**

Gout and diabetes mellitus type 2 (DM) frequently co-exist. The pharmacological effects of metformin may include anti-inflammatory and urate lowering effects. The objective of this study was to test these effects in patients with gout starting uric acid lowering treatment (ULT) in secondary care.

**Methods:**

Retrospective cohort study including patients with gout and DM starting ULT. Differences in the incidence density of gout flares, proportion of patients reaching target sUA in the first six months after starting ULT, and difference in mean allopurinol dose at sUA target were compared between users of metformin and users of other or no anti-diabetic drugs (control group). Correction for confounding was applied.

**Results:**

A total of 307 patients were included, of whom 160 (52.1%) used metformin. The incidence of flares was 1.61 and 1.70 in the first six months for respectively the metformin group and control group. The incidence rate ratio for gout flares was not significant (0.95, 95% CI 0.78 to 1.14). At six months, 62.8% and 54.9% reached target sUA in the metformin and control group respectively, corrected odds ratio of 1.09 (95% CI 0.66 to 1.80). There was no difference in mean allopurinol dose at sUA target 266 mg for metformin users and 236 mg for the control group, difference 30 mg (95% CI − 4.7 to 65.5).

**Conclusions:**

In conclusion we could not confirm a clinically relevant anti-inflammatory or urate lowering effect of metformin in patients starting ULT treatment and receiving usual care flare prophylaxis.

## Introduction

Gout is one of the most prevalent inflammatory rheumatic diseases worldwide and its prevalence is increasing [1]. Drug treatment of gout focuses on treating acute gout flares with anti-inflammatory drugs and reducing serum uric acid (sUA) levels with urate lowering therapy (ULT) [2]. Patients with gout often have comorbidities, like diabetes mellitus (DM) which is present in a quarter of patients with gout [3].

Metformin is the first-choice medication for patients with type 2 DM. Recently, it has been suggested that metformin also has anti-inflammatory effects in gout. These effects are mainly mediated by 5’Adenosine Monophosphate-activated Protein Kinase (AMPK) through different mechanisms [4]. A downstream target of AMPK is mammalian target of rapamycin (mTOR), which is one of the biological mechanisms involved in the process of inflammation [5, 6]. Metformin has shown to reduce mTOR signalling in cells contacted with monosodium urate crystals [7]. A small retrospective study found that diabetic gout patients who used metformin and allopurinol had a significantly lower number of gout attacks, compared to diabetic gout patients who used allopurinol alone [7].

In addition to putative anti-inflammatory effects, metformin is believed to have a sUA lowering effect by improving insulin sensitivity. There are two proposed mechanisms for this effect. First, urinary uric acid clearance appears to increase with higher insulin sensitivity, leading to a decrease in sUA [8–10]. Second, insulin resistance causes lipolysis which leads to higher levels of free fatty acids, that are eventually metabolised into uric acid [9, 11]. This effect was indeed found in a small controlled intervention study with metformin in patients with gout who did not use ULT [12].

In conclusion, there is some evidence on the anti-inflammatory and sUA lowering effects of metformin, but relevance for clinical practice is unknown [4]. We therefore conducted this retrospective cohort study, to examine whether metformin has a relevant anti-inflammatory and sUA-lowering effects in a clinical practice context.

## Methods

### Study design

We conducted a retrospective cohort study in secondary care setting. Eligible patients were included from the rheumatology departments of three hospitals (Sint Maartenskliniek, Rijnstate and Radboudumc) in The Netherlands. Data was collected from electronic health records, including patient-, disease- and treatment characteristics. The local ethics committee (Commissie Mensgebonden Onderzoek regio Arnhem-Nijmegen, 2018-4692) assessed the study and provided exemption, as ethical approval for this type of study is not required under Dutch law.

### Participants

The retrospective cohort included patients ≥ 18 years with the diagnosis gout and at least six months follow-up. Eligible for this study were patients with a diagnosis of DM, a first prescription of ULT after inclusion in the cohort and at least six months follow-up after initiation of ULT. Metformin use was operationalised as prescription coverage of metformin in any dose for at least 80% (145 days) of the six months follow-up. This cut-off point was chosen in line with the minimal use of 80% to be adherent to medication [13]. Patients without a minimum prescription coverage of 80% were excluded from the study. DM patients with other or no medication were placed in the control group.

### Outcome measures

#### Anti-inflammatory effect

To evaluate the anti-inflammatory effect of metformin, we assessed the incidence rate ratio (IRR) of gout flares in the first 6 months after start of ULT. We defined gout flares as a clinical diagnosis of gouty arthritis by the physician, based on physical examination and laboratory inflammation parameters when available. In addition, flares in the period before consultation and reported by patients at the consultation were included as total of flares over the period between each consultation. Incidence of gout flares during the first six months of start ULT was calculated by attributing the number of flares reported during a consultation to the time since the last consultation. Total number of flares divided by sum of person-time was used to calculate the incidence density (ID) over the six month period of interest. When no information was reported, it was assumed that no flares had occurred.

#### Serum uric acid lowering effect

To evaluate the sUA lowering effect of metformin, we assessed sUA levels at baseline, sUA change over the first six months, the proportion of patients who reached sUA target (< 0.36 mmol/l) within six months and the dose of allopurinol at sUA target. sUA levels were collected from their respective lab files in the electronic health record. The last known sUA measurement was used for the proportion of patients who reached target within 6 months, if there was no measurement available in the last month, but available within two weeks after 6 months, we used the latter one. Patients were excluded from these specific analyses if there were no sUA measurements available within this period. A sUA target of < 0.36 mmol/l was used, following the EULAR/ACR guidelines [2]. Dose of ULT was collected from the medication sheets in the electronic health record.

### Statistical analysis

No formal sample size calculation was made as a convenient sample was used. All comparisons were made for metformin users compared to the control group as reference. Baseline characteristics were evaluated using two-sample t-test or Mann–Whitney U test, depending on distribution for continuous variables. For categorical variables chi-square test or Fisher’s exact test were used. To evaluate the difference in ID of flares in the first 6 months after starting ULT, Poisson regression was used. At first, in univariate analysis all variables which changed the estimate for more than 10% were selected as confounders in the full analysis. These included age, alcohol use, colchicine use, usage of anti-inflammatory drugs, prednisone use, renal impairment, sUA at baseline, crystal proven gout, insulin use and presence of tophi. Difference in sUA levels at baseline was evaluated by linear regression. The full linear model included renal impairment, diuretic use, insulin use and crystal proven gout as confounders. ULT dose at time of reaching target was evaluated by linear regression as well. There were no confounders included. A linear mixed model with random intercept was used to compare the course of sUA levels over the first 6 months. This model included sUA at baseline and renal impairment as confounders. To compare the number of patients that reached or did not reach sUA target levels, logistic regression was used. This model included renal impairment, use of diuretics and sUA at baseline as confounders. Statistical analyses were performed in STATA/IC v 13.1.

## Results

Of a total of 1401 naive ULT starters with six months follow-up, 307 (22%) patients with DM were included in this study (Table 1). The metformin group consisted of 160 patients and the control group 147. Metformin users were somewhat younger and had a better renal function compared to non-metformin users. Most patients started with allopurinol as ULT.

**Table 1:** Baseline and disease characteristic

Baseline characteristics	Metformin group (n = 160)	Control group (n = 147)	*P* value
Age (years) Median (IQR)	70.6 (65.1–77.2)	74.4 (66.7–79.6)	**0.0187**
Male gender (%)	114 (71.3)	104 (70.8)	0.923
BMI (kg/m^2^) Median (IQR)*	30.1 (27.3–33.2)	31.2 (26.2–35.7)	0.8495
Alcohol use (%)	69 (43.1)	59 (40.1)	0.248
Comorbidities**			
Hypertension (%)	109 (68.1)	92 (62.6)	0.308
Hypercholesterolemia (%)	35 (21.9)	31 (21.1)	0.867
Kidney stones (%)	7 (4.4)	10 (6.8)	0.353
Renal impairment (%)	37 (23.1)	63 (42.9)	**0.000**
Serum uric acid baseline (mmol/l) Mean (± SD)	0.54 (± 0.12)	0.56 (0.12)	0.1339
Renal function, eGFR (ml/min/1.73 m^2^) Median (IQR)	60 (48–70)	50 (34–68)	**0.0008**
Medication			
Diuretics (%)	111 (69.4)	93 (63.3)	0.257
Insulin (%)***	32 (20)	39 (26.5)	0.175
Other oral diabetics (%)***	75 (46.9)	56 (38.1)	0.1194
Number of involved joints			0.396
Mono articular disease: 1 joint (%)	33 (20.8)	22 (15)	
Oligo articular disease**:** 2–4 joints (%)	77 (48.4)	79 (53.7)	
Poly articular disease: > 4 joints (%)	49 (30.8)	46 (31.3)	
MTP-1 involved (%)	100 (70.4)	95 (74.8)	0.422
Tophi (%)	53 (33.1)	57 (38.8)	0.302
Crystal-proven gout (%)	117 (73.1)	119 (81)	0.104
Erosions (%)	26 (16.3)	30 (20.4)	0.346
ULT started			0.450
Allopurinol (%)	156 (97.5)	144 (98)	
Benzbromarone (%)	4 (2.5)	2 (1.4)	
Febuxostat (%)	0	1 (0.7)	
Start dose allopurinol (mg/day) median (IQR)	100 (100–100)	100 (100–100)	0.3469
Colchicine use (%)	115 (71.9)	102 (69.4)	0.632

Two-sample t-test or Mann–Whitney U test, depending on distribution for continuous variables. For categorical variables chi-square test or Fisher’s exact test

BMI = body mass index (kg/m^2^), eGFR = estimated glomerular filtration rate (ml/min/1.73m^2^), MTP = (metatarsophalangeal joint). ULT = urate lowering therapy

*> 50% of data is missing

**As stated in the electronic patient record

***Some patients used both insulin and other oral diabetics

### Gout flares

In the metformin group, the ID of gout flares in the first six months after starting ULT was 1.61 (95% CI 1.22 to 2.01), compared to 1.70 (95% CI 1.38 to 2.01) in the control group. The adjusted incidence rate ratio (IRR) was 0.95 (95% CI 0.78–1.14) (for unadjusted estimates see Table 2).

**Table 2:** Uncorrected and corrected analyses per outcome measure

	Outcome	Confounders
*Gout flares*		
Uncorrected	0.95 IRR (95% CI 0.80–1.13)	
Corrected	0.95 IRR (95% CI 0.78–1.14)	Age, alcohol use, colchicine use, prednisone use, use anti-inflammatory drugs, renal impairment, sUA at baseline, crystal proven gout, presence of tophi and insulin use
*sUA levels baseline*		
Uncorrected	− 0.02 mmol/l difference (95% CI − 0.05–0.01)	
Corrected	− 0.02 mmol/l difference (95% CI − 0.04–0.01)	Renal impairment, diuretic use, insulin use and crystal proven gout
*sUA levels over 6 months*		
Uncorrected	− 0.02 mmol/l difference (95% CI − 0.04–0.00)	
Corrected	− 0.01 mmol/l difference (95% CI − 0.02–0.01)	sUA at baseline and renal impairment
*Reaching target sUA*		
Uncorrected	1.39 OR (95% CI 0.87–2.20)	
Corrected	1.09 OR (95% CI 0.66–1.80)	Renal impairment, history of kidney stones sUA at baseline, insulin use and use of diuretics
*Dose at target allopurinol*		
Uncorrected	30.4 mg difference (95% CI − 4.7–65.5)	

*sUA* serum uric acid, *IRR* incidence rate ratio, *OR* odds ratio

### SUA levels

**Fig. 1:**
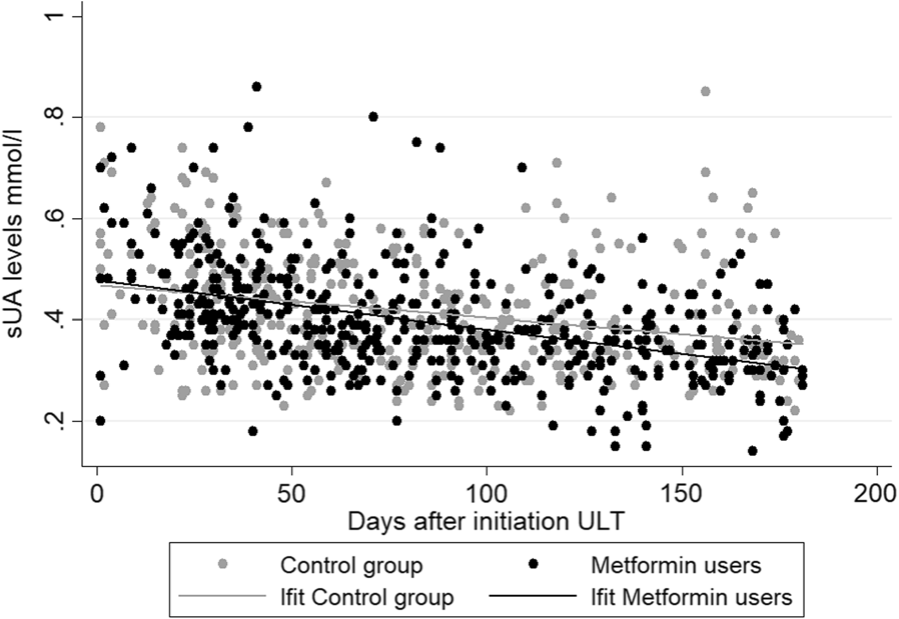
Change in sUA levels over the first 6 months after initiation ULT. *Lfit gives an indication of the decrease in sUA over time in both groups

Mean sUA levels at baseline were 0.54 mmol/l and 0.56 mmol/l, for the metformin group and control group, respectively. Adjusted linear regression showed that sUA levels at baseline did not differ between both groups (difference − 0.02, 95% CI − 0.04 to 0.01) (unadjusted estimates see Table 2). Mean sUA levels at last known measurement before six months were 0.35 mmol/l and 0.38 mmol/l, for the metformin group and control group, respectively. As illustrated in Fig. 1 we found no differences in change over six months in sUA levels between both groups (adjusted difference − 0.01, 95% CI − 0.02 to 0.01) (unadjusted estimates see Table 2).

### Target serum uric acid

Within the first six months, 62.8% of the metformin group had reached target sUA levels compared to 54.9% in the control group (adjusted odds ratio 1.09 (95% CI 0.66–1.80)) (unadjusted estimates see Table 2). Mean daily dosages of allopurinol at target were 266 (± 121) and 236 (± 100) for the metformin group and control group, respectively. Linear regression showed no significant between group differences (difference 30 mg/day, 95% CI − 4.7 to 65.5).

## Discussion

We did not observe a relevant anti-inflammatory or sUA lowering effect of metformin during the first six months after starting ULT in a real-world setting. Although these effects of metformin are supported by pharmacological and empirical evidence, several contextual factors can lead to a null effect when treating gout patients in a real-world setting.

Firstly, the anti-inflammatory effect of metformin might be too weak to have a clinically relevant contribution in gout treatment in a phase where strong anti-inflammatory treatments like colchicine are prescribed as prophylactic treatment [3]. Another explanation for the lack of difference in gout flares in this study is the effect of other possible variables that interfere with the proposed anti-inflammatory mechanism of metformin, for example state of diabetes regulation. Poorly controlled diabetes is described to decrease the risks of gout flares in some studies [14, 15]. This suggested mechanism in diabetes mellitus might counteract the possible effect of metformin, however we did not have the data to correct for this possible mechanism.

The lack of sUA lowering effect of metformin might be driven by differences in study context and design. The study by Barskova et al. [12] was a small intervention study with metformin in which the included patients did not use ULT. In our study all patients started ULT. Also, in our study only prevalent metformin users were included. It is therefore possible that through index event bias [16] our sample disproportionally included patients in whom metformin did not have a sUA lowering effect, or not enough to prevent the development of gout. However, index event bias would also reduce the proportion of DM patients and metformin users in our cohort, but with 22% DM patients of which 52% used metformin our cohort stays well within the expected ranges [3, 17]. Furthermore, other anti-diabetic medication may have this sUA lowering effect in gout as well, thus resulting in a net null result. Whether this effect is unique for metformin has indeed not been tested. Of note, previous studies have shown that even drugs within the same class can have different off-target effects, for example in a study comparing losartan and irbesartan, only losartan showed a sUA lowering effect in patients with gout [18].

This retrospective study might have some general limitations, such as underreporting of gout flares and a possibility of double reported flares. However, firstly we assume that this would be the case in both groups and probably should not result in a biased between group difference, secondly our flare rate is comparable with other studies [19, 20]. Also, we had no data on the type of DM. However, it is likely that most patients have type 2 DM since this accounts for 90 to 95% of all DM, and gout is mainly associated with type 2 DM [3, 21]. Also, we had no data on the state of diabetes regulation, including HbA1c levels, which may interact with the risk of gout flares as well [14, 15]. In the non-metformin group mean age was slightly higher and renal function lower, resulting in confounding by indication. However, our analyses were corrected for these differences when necessary.

Strengths of this study include the considerable sample size, resulting in adequate precision while excluding any relevant effect considering the confidence intervals, and correction for confounders. Due to the non-limiting inclusion criteria, multi-centre data collection and a prevalence of DM in the cohort within the expected range, the generalisability of the study seems solid. Also, the uricosuric effect of metformin was assessed using different outcome measures, including correction for second order effects such as differences in ULT use.

In conclusion, although pharmacological effects of metformin probably include anti-inflammatory and urate lowering effects, we could not confirm a clinically relevant effect in patients starting ULT treatment and receiving usual care flare prophylaxis.

## Data Availability

The datasets generated and/or analysed during the current study are not publicly available due to ongoing study activity but are available from the corresponding author on reasonable request.

## Abbreviations

sUA: Serum uric acid
ULT: Urate lowering therapy
DM: Diabetes mellitus
AMPK: 5′Adenosine Monophosphate-activated Protein Kinase
mTOR: Mammalian target of rapamycin
ID: Incidence density
IRR: Incidence rate ratio
OR: Odds ratio
